# Deciphering Amblyopia: Epidemiological Insights From a Pediatric Study

**DOI:** 10.7759/cureus.81818

**Published:** 2025-04-07

**Authors:** Kavita R Bhatnagar, Falguni Roy, Kirti Jaisingh, Nikhil Agrawal, Pankaj Bhardwaj, Pankaja Raghav

**Affiliations:** 1 Ophthalmology, All India Institute of Medical Sciences, Jodhpur, Jodhpur, IND; 2 Community Medicine & Family Medicine, All India Institute of Medical Sciences, Jodhpur, Jodhpur, IND

**Keywords:** amblyopia, anisometropia, astigmatism, childhood blindness, non-ocular risk factors, ocular risk factors, refractive amblyopia, screening programs, stimulus deprivation, strabismus

## Abstract

Background

This study aimed to determine the prevalence and risk factors associated with amblyopia among preschool and school-going children aged 3-12 years.

Methodology

A cross-sectional, observational study was conducted on 1,683 preschool and school-going children in western India. They were divided into three groups (group A: 3-5 years, group B: 6-9 years, and group C: 10-12 years). All participants underwent comprehensive ophthalmological evaluations to identify ocular causes of amblyopia such as refractive errors, strabismus, and stimulus deprivation. Non-ocular risk factors were assessed through detailed parental questionnaires addressing prenatal, perinatal, and postnatal histories.

Results

Of the children screened, 5.88% (n = 99) were diagnosed with refractive errors, and 1.66% (n = 28) were diagnosed with amblyopia. Among amblyopic children, refractive amblyopia was the most prevalent at 71.43% (n = 20, p < 0.0001), followed by strabismic amblyopia (21.43%, n = 6) and stimulus deprivation amblyopia (7.14%, n = 2). Astigmatism was found to be most amblyogenic in cases of both unilateral and bilateral amblyopia, though hypermetropic amblyopic children formed the majority of the refractive amblyopia population. The majority of children had moderately severe amblyopia across all age groups. Significant non-ocular risk factors included pre-obese maternal body mass index at conception (p < 0.001) and low birth weight (p < 0.001). Although preterm birth also emerged as a major risk factor, the results were not significant (p = 0.063).

Conclusions

Amblyopia in children can be attributed to both modifiable ocular and non-ocular risk factors. It is one of the most common reversible causes of childhood blindness. Early identification and intervention are essential for optimal visual outcomes, highlighting the importance of initiating amblyopia screening programs starting at preschool ages.

## Introduction

Amblyopia, often referred to as “lazy eye,” is a visual disorder characterized by a unilateral and, less commonly, bilateral reduction of best-corrected visual acuity that is not attributable solely to any apparent structural abnormality of the eye or visual pathway [1,2]. It remains a significant pediatric concern due to its potential to persist into adulthood if not adequately addressed during childhood. Global prevalence rates of amblyopia vary widely, affecting between 0.2% and 6.2% of children worldwide, underscoring its relevance across diverse populations [3].

The etiology of amblyopia is multifactorial, encompassing both ocular and non-ocular risk factors. Ocular contributors include strabismus, sensory deprivation in the form of cataract and central corneal opacities, anisometropia, and high refractive errors, which disrupt normal visual development, leading to amblyopia [3-5]. Additionally, congenital nasolacrimal duct obstruction is recognized for its role in inducing amblyopia through prolonged epiphora and intermittent discharge, which result in visual blur [6]. Non-ocular risk factors extend beyond the eye itself, including prematurity, developmental delays, being small for gestational age, or having a first-degree relative with amblyopia. Environmental influences, such as maternal substance use during pregnancy, have also been linked to an increased risk of developing amblyopia or strabismus [7].

The imperative for early detection and intervention cannot be overstated. The success of amblyopia treatment is contingent upon several factors, including the cause, the age at which the amblyogenic stimulus began, the age of beginning amblyopia therapy, the type of amblyopia therapy chosen, the severity and duration of the condition, previous interventions, adherence to treatment, and the presence of coexisting pathologies [8]. Despite this knowledge, there remains a significant gap in the literature concerning the comprehensive identification of both ocular and non-ocular risk factors for amblyopia, particularly in the Indian subcontinent.

This study aims to fill this gap by elucidating the prevalence and a broad spectrum of risk factors associated with amblyopia among children aged 3-12 years in the western part of Rajasthan. By doing so, it seeks to underline the critical need for systematic screening and comprehensive ophthalmological evaluations, aiming to enhance early detection rates and improve visual outcomes for affected children.

## Materials and methods

This prospective, observational study was performed in various schools of Rajasthan, from January 2021 to December 2021, targeting preschool and school-going children, covering both government and private schools. Approval was granted by the Institutional Ethics Committee, All India Institute of Medical Sciences, Jodhpur (AIIMS Jodhpur) (reference number: AIIMS/IEC/2021/3361). The protocol adhered to the principles of the Declaration of Helsinki.

After obtaining permission from the district health administration, schools were randomly selected for participation. Written informed consent was secured from all participants’ parents or guardians before any assessment. All children aged 3-12 years who underwent comprehensive ophthalmological screening and whose parents gave consent were included in the study. Any child with an organic eye disease or uncooperative behavior during examination was excluded from the study. The children participating in the study were divided into three age groups. Group A included children aged 3-5 years, group B included children aged 6-9 years, and group C included children aged 10-12 years.

Comprehensive eye examinations were conducted in the school premises by a team of trained ophthalmologists and an optometrist. All children were assessed for uncorrected and best-corrected visual acuity, ocular alignment, ocular motility, intraocular pressure (IOP), and any abnormality of anterior or posterior segments, including adnexal tissues. Visual acuity testing methods were age-appropriate, utilizing Tumbling E and pictorial vision charts for younger children and Snellen’s charts for older children. Cycloplegic refraction was performed using 1% cyclopentolate eye drops instilled three times per eye, with retinoscopy performed 25 minutes after the last instillation in all children. Detailed squint evaluation was performed in cases of ocular misalignment. IOP was measured digitally in all children. Anterior segment examination was done using a direct ophthalmoscope, and the posterior segment was examined using an indirect ophthalmoscope. All children diagnosed with refractive errors were prescribed glasses and asked to follow up in the outpatient department (OPD) of the Ophthalmology Department of AIIMS Jodhpur. Children with amblyopia and other ocular anomalies were also similarly called to OPD for further testing and treated accordingly.

Standard definitions for various refractive errors and subtypes of amblyopia were used. Refractive errors were classified as myopia, hypermetropia, and astigmatism. These were further categorized as mild, moderate, or severe based on specific criteria. Myopia was classified as mild (<3.0 D), moderate (3.25-6.0 D), and severe (>6.0 D); hypermetropia as mild (<2.0 D), moderate (2.25-5.0 D), and severe (>5.0 D); and astigmatism as mild (<1.0 D), moderate (1.0-2.0 D), and severe (>2.0 D) [9-11].

Amblyopia was defined as a difference in the best-corrected visual acuity between the two eyes of 2 or more Snellen lines/equivalent measure on the pictorial or Tumbling E chart in children aged less than four years, in the absence of any organic lesion that could result in a decrease in vision. In cases of bilateral amblyopia, it was defined as a best-corrected visual acuity of less than 6/12 bilaterally on the Snellen’s chart/equivalent measure on the pictorial or Tumbling E chart in children aged less than four years, in the absence of any organic lesion that could result in a decrease in vision. Amblyopia severity was defined based on visual acuity in the affected eye: mild (6/9 to 6/12), moderate (6/12 to 6/36), and severe (worse than 6/36) [2].

Amblyopia was further categorized as refractive, strabismic, and stimulus deprivation amblyopia [12,13]. Refractive/ametropic amblyopia was diagnosed when hypermetropia was >4.00 D, myopia >6.00 D, and astigmatism >2.50 D bilaterally with no related strabismus or ocular pathology. Anisometropic amblyopia was diagnosed when there was 1.00 D or more in spherical equivalent for hypermetropia, 3.00 D or more for myopia, and 1.50 D or greater difference in astigmatism in any meridian between the two eyes. Strabismic amblyopia was defined as amblyopia in the presence of heterotropia at a distance and/or near, or a history of strabismus surgery, and in the absence of refractive error significant to cause heterotropia. All cases with significant anisometropia potentially able to cause heterotropia were included under refractive amblyopia. Deprivation amblyopia included patients with known documented cases of sensory deprivation (cataract, ptosis, or other media opacities) with no primary heterotropias or refractive errors that could be causally related to amblyopia.

A detailed proforma was used to collect data on each child’s birth history, maternal health factors, environmental exposures during pregnancy, intranatal and postnatal complications, presence of any ocular pathology, and family history of amblyopia. Preterm birth was defined as a gestation period less than 37 weeks. Low birth weight was categorized as a birth weight under 2,500 g, whereas normal birth weight ranged from 2,500 g to 4,000 g. The body mass index (BMI) of the mother was calculated using the following standard formula: weight in kilograms divided by the square of height in meters, with categories for underweight (<18.5 kg/m^2^) and overweight (>25 kg/m^2^).

Data were entered into Microsoft Excel and analyzed using SPSS version 25 (IBM Corp., Armonk, NY, USA). Descriptive statistics, such as counts and percentages, were used for nominal variables and analyzed using chi-square or Fisher’s exact tests. Ordinal variables were described using medians and interquartile ranges, and analyzed using the Mann-Whitney U test. Statistical significance was set at a p-value less than 0.05.

## Results

A total of 1,683 children participated in the study and were categorized into three groups. Group A comprised 307 children, group B included 547 children, and group C had 829 children. The mean age of the participants was 8.92 ± 2.20 years. The cohort demonstrated a male predominance, with males constituting 63.81% (n = 1,074) and females 36.19% (n = 609) (Table 1).

**Table 1:** Gender distribution of the total population and amblyopic children.

Gender	Frequency of amblyopia (n = 28)	Total (n = 1,683)	P-value
Male	15 (13.96%)	1,074 (63.81%)	0.320
Female	13 (21.35%)	609 (36.19%)	

Refractive errors were identified in 5.88% (n = 99) of the children, with the highest prevalence in group C at 6.27% (n = 52), followed by group B at 5.85% (n = 32), and group A at 4.88% (n = 15). However, the difference was insignificant among the three groups (p = 0.316) (Table 2).

**Table 2:** Age-wise prevalence of refractive errors.

Age groups	Frequency of refractive error (n = 99)	Total (n = 1,683)	P-value
3–5 years	15 (4.88%)	307 (18.24%)	0.316
6–9 years	32 (5.85%)	547 (32.50%)	
10–12 years	52 (6.27%)	829 (49.26%)	

Myopia was the most common refractive error in our study, accounting for 45.45% (n = 45), followed by hypermetropia (40.40%) and astigmatism (14.14%). Astigmatism was most prevalent in the younger age group, and its frequency decreased with increasing age (Table 3).

**Table 3:** Distribution of different refractive errors among all three age groups.

Types of refractive errors (n = 99)	3–5 years (n = 15)	6–9 years (n = 32)	10–12 years (n = 52)	P-value
Myopia (n = 45; 45.45%)	6 (40%)	17 (53.12%)	22 (42.31%)	0.334
Hypermetropia (n = 40; 40.40%)	5 (33.3%)	11(34.4%)	24 (46.16%)	
Astigmatism (n = 14; 14.14%)	4 (26.7%)	4 (12.5%)	6 (11.5)	

The prevalence of amblyopia was 1.66% (n = 28), with no significant gender preference (p = 0.32) (Table 1). The prevalence of amblyopia across the groups was also statistically insignificant (p = 0.95), with group A at 1.63% (n = 5), group B at 1.83% (n = 10), and group C at 1.57% (n = 13) (Table 4).

**Table 4:** Prevalence of amblyopia in different age groups.

Age groups	Prevalence of amblyopia (n = 28; 1.66%)	Total	P-value
3–5 years	5 (1.63%)	307 (100%)	0.950
6–9 years	10 (1.83%)	547 (100%)	
10–12 years	13 (1.57%)	829 (100%)	

In our study, unilateral amblyopia was more common than bilateral amblyopia, as seen in 57.4% (n = 16) of the children compared to 42.86% (n = 12) of bilateral amblyopia; however, the difference was statistically insignificant (p = 0.45) (Table 5).

**Table 5:** Laterality of amblyopia.

Laterality of amblyopia	Unilateral	Bilateral	Total	P-value
Number of children	16 (57.4%)	12 (42.86%)	28	0.446

While evaluating the subtypes of amblyopia, refractive amblyopia was the most common subtype, accounting for 71.43% (n = 20) of cases (p < 0.001), followed by strabismic amblyopia at 21.43% (n = 6), and deprivation amblyopia at 7.14% (n = 2) (Table 6).

**Table 6:** Types of amblyopia.

Type of amblyopia (n = 28)	Number of children	P-value
Refractive	20	<0.001
Strabismic	6	
Stimulus deprivation	2	

In refractive amblyopia, anisometropia was a notable risk factor in 40% (n = 8) of amblyopic children (p = 0.308). However, there was no significant correlation between the severity of anisometropia and the depth of amblyopia (p = 0.196). Others had ametropic amblyopia, with hypermetropia constituting the maximum number of cases at 6 (30%), followed by equal incidence of myopia and astigmatism at 15% each (Tables 7, 8).

**Table 7:** Types of refractive amblyopia.

Type of refractive amblyopia (n = 20)	Number of children	P-value
Hypermetropia	6 (30%)	0.308
Myopia	3 (15%)	
Astigmatism	3 (15%)	
Anisometropia	8 (40%)	

**Table 8:** Severity of amblyopia.

Severity of anisometropia in amblyopic children (n = 8)	Number of children	P-value
Mild	1	0.196
Moderate	2	
Severe	5	

In cases with strabismic amblyopia (nine children), there was no significant difference between the type of deviation causing amblyopia (p = 0.097) (Table 9).

**Table 9:** Type of strabismus in strabismic amblyopic children.

Type of strabismus in amblyopic children (n = 9)	Number of children (n = 9)	P-value
Esotropia	4 (45.5%)	0.097
Exotropia	5 (55.5%)	
Vertical deviation	0 (0%)	

In our study, moderate amblyopia was the most frequent in almost all age groups. There was no significant correlation of severity of amblyopia with age, with p-values of 0.44 and 0.64 in the right eye and the left eye, respectively. Eight children had severe amblyopia involving the right eye, and nine children had severe amblyopia in the left eye, with a few cases having severe bilateral amblyopia (Table 10).

**Table 10:** Severity of amblyopia in various age groups.

Severity of amblyopia		3–5 years	6–9 years	10–12 years	Total	P-value
Right eye	Mild	0 (0%)	0 (0%)	2 (25%)	2 (10%)	0.436
	Moderate	2 (50%)	4 (50%)	4 (50%)	10 (50%)	
	Severe	2 (50%)	4 (50%)	2 (25%)	8 (40%)	
Left eye	Mild	0 (0%)	0 (0%)	1 (9.10%)	1 (4.76%)	0.639
	Moderate	4 (80%)	2 (40%)	5 (45.45%)	11 (52.38%)	
	Severe	1 (20%)	3 (60%)	5 (45.45%)	9 (42.86%)	

However, astigmatism was found to be most amblyogenic in both cases of unilateral and bilateral amblyopia (Table 11).

**Table 11:** Types and severity of refractive errors causing amblyopia.

Refractive error		Frequency (n = 1,683)	Unilateral amblyopia	Bilateral amblyopia	P-value
Right eye	Emmetropia	1,589	0 (0%)	0 (0%)	0.670
	Mild myopia	24	0 (0%)	0 (0%)	
	Moderate myopia	10	0 (0%)	0 (0%)	
	Severe myopia	10	2 (20%)	3 (30%)	
	Mild hypermetropia	11	4 (36.36%)	0 (0%)	
	Severe hypermetropia	29	3 (10.34%)	5 (17.24%)	
	Mild astigmatism	0	0 (0%)	0 (0%)	
	Moderate astigmatism	10	4 (40%)	1 (10%)	
	Severe astigmatism	6	3 (50%)	2 (33.3%)	
Left eye	Emmetropia	1,583	0 (0%)	0 (0%)	0.556
	Mild myopia	23	0 (0%)	0 (0%)	
	Moderate myopia	15	3 (33.3%)	0 (0%)	
	Severe myopia	7	1 (14.3%)	3 (42.86%)	
	Mild hypermetropia	8	0 (0%)	0 (0%)	
	Severe hypermetropia	33	1 (3.03%)	5 (15.15%)	
	Moderate astigmatism	9	2 (22.22%)	1 (11.11%)	
	Severe astigmatism	5	2 (40%)	2 (40%)	

Various non-ocular risk factors that have been shown in some studies to be amblyogenic were also studied, including demographic details of the mother at conception and the infant at birth. The mean age of mothers at conception was 22.30 ± 4.30 years, with a mean weight of 55.60 ± 6.41 kg and a mean height of 153.76 ± 10.82 cm. The average BMI was 24.39 ± 4.46 kg/m^2^. A highly significant association between amblyopia and mothers' BMI was seen (p < 0.001). Most of the children with amblyopia had mothers with BMI in the pre-obese/overweight category at the time of conception (25-29.9 kg/m^2^). Preterm infants were more susceptible to developing amblyopia, though the results were not statistically significant. Low birth weight was also significantly associated with increased risk of amblyopia in later life (p < 0.001). On evaluating postnatal complications, the odds of amblyopia were much lower in all complication groups compared to the no complications group (Table 12).

**Table 12:** Non-ocular risk factors of amblyopia. BMI: body mass index; VLBW: very low birth weight; LBW: low birth weight

Non-ocular risk factors		Frequency of children	Relative risk/Odds ratio	P-value
Maternal BMI (kg/m^2^)	<18.5	1 (3.57%)	Not analyzed	<0.001
	18.5–24.9	11 (39.29%)		
	25–29.9	14 (50%)		
	30–34.5	2 (7.14%)		
	>35	0 (0%)		
Birth weight	VLBW (<1,500 g)	2 (7.14%)	0.20/0.196	<0.001
	LBW (<2,500 g)	16 (57.14%)	1.60/1.624	
	Normal	10 (35.71%)	1.00/1.000	
	Overweight	0 (0%)	0.00/0.000	
Postnatal complications	No complications	20 (71.43%)	1.00/1.00	<0.001
	Birth asphyxia	1 (3.57%)	0.05/0.048	
	Respiratory distress	5 (17.86%)	0.25/0.241	
	Neonatal jaundice	2 (7.14%)	0.10/0.096	
Gestational age	Moderately preterm	10 (35.71%)	Not analyzed	0.063
	Late preterm	4 (14.29%)		
	Term	14 (50%)		

## Discussion

Amblyopia is one of the most common causes of reversible childhood blindness. Early detection and intervention in the form of just refractive correction alone can successfully treat a large part of the amblyopic population. In our study, which involved screening 1,683 children, amblyopia was detected in 1.66% of cases. This prevalence is consistent with findings from studies conducted by Kalikiyavi et al. and Gupta et al., where similar prevalence rates were observed [5,14]. However, studies by Murthy et al. and Anjaneyulu et al. reported higher rates at 4.4% and 6.6%, respectively [15,16]. Generally, the prevalence of amblyopia reported in the literature ranges from 0.8% to 3%, with variations likely due to differences in age ranges assessed and the diversity of study populations.

In our research, refractive amblyopia was identified as the most common ocular cause, affecting 71.43% of amblyopic children, followed by strabismus and deprivation amblyopia. These findings align with the MEPEDS study, which reported refractive amblyopia in 78% of cases [17]. Moreover, strabismic amblyopia may be detected and treated earlier due to the visible deviation of the eyes. Anisometropia was a notable risk factor in our study, present in 40% of the children with amblyopia. This finding is consistent with studies by Mocanu et al., Ganekal et al., and Pascual et al., all of which highlighted anisometropic amblyopia as a prevalent refractive risk factor [18-20].

Strabismus was observed in 21.43% of amblyopic cases in our study. Although not the most prevalent cause, it remains a significant risk factor for amblyopia, particularly noted in studies involving multiethnic groups such as African Americans and Hispanics [17]. Our study showed severe astigmatism to be the most common type of refractive error causing unilateral and bilateral amblyopia (50% in the right eye and 40% in the left eye for unilateral amblyopia, and 33.33% in the right eye and 40% in the left eye for bilateral amblyopia). Studies by Ganekal et al and Hendler et al. echoed similar findings [19,21]. Pascual et al. reported that hyperopia >2 D, astigmatism >1 D, and anisometropia >0.5 D are significant risk factors for refractive amblyopia [20].

Regarding the severity of amblyopia, the majority of affected eyes in our study exhibited moderate amblyopia in approximately half of the patients across all age groups. Severe amblyopia was more commonly seen across older age groups compared to the 3-5-year age group, signifying an increasing probability of amblyopia with age if left untreated. This trend of increasing severity of amblyopia mirrored the study by Donahue, who showed a similar relationship between patient age and amblyopia [22]. However, this distribution of severity contrasts with the study by Sapkota et al., which found a predominance of mild to moderate amblyopia in 60% of cases [23]. Ganekal et al. also showed a higher percentage of moderate amblyopia [19]. This could be attributed to the higher prevalence of refractive amblyopia than strabismic amblyopia in our study, which causes less severe amblyopia compared to strabismic amblyopia.

Significant associations with amblyopia were also found concerning low birth weight (p < 0.001), similar to the findings by Robaei et al, who underscored the links between these factors and amblyopia [24]. Maternal BMI before conception also showed a significant correlation with amblyopia, particularly within the pre-obese range (25-29.9 kg/m^2^), a finding echoed by Mocanu et al. [18]. Amblyopia is a neurodevelopmental disorder. Maternal obesity can lead to various antenatal and perinatal complications, which can further affect the neural development of these babies. However, this is just a speculation, and the exact mechanisms need to be determined by further studies.

Contrary to expectations, postnatal complications appeared to be associated with a lower risk of amblyopia. This suggests that factors such as increased medical attention in complicated births may reduce amblyopia risk, as such children were more likely to receive early interventions. However, the literature is quite scarce regarding these. A study by Pan et al. did find a significant correlation of amblyopia with children who had APGAR scores less than 7 [25].

Our study had several limitations, including potential inaccuracies in the parental reporting of factors such as the mother’s preconception weight and smoking history or a family history of amblyopia. These could have caused a potential recall bias. Additionally, the study’s population size and the exclusion of children unable to complete visual acuity testing or absent on screening day may have introduced selection bias. The absence of some children during follow-up appointments also potentially affected the robustness of our findings. Moreover, the study was conducted among healthy children going to normal schools only. It excluded children with severe ischemic insult, which could have modified the relationship of amblyopia with perinatal complications. Being a cross-sectional study, the exact causality of non-ocular risk factors could not be determined. However, our study is one of the very few studies that have studied the prevalence of amblyopia in the preschool age group and helped children gain access to early treatment options. Such early interventions are instrumental in building a healthy future not only for such children but also for our country.

## Conclusions

This study provides valuable insights into the prevalence and risk factors associated with amblyopia among children aged 3-12 years in western Rajasthan. The prevalence of amblyopia was determined to be 1.66%, which aligns with existing research, suggesting consistency across different geographic and demographic settings. The study highlights refractive amblyopia as the most prevalent cause, with significant contributions from anisometropia and strabismus. We also identified several key non-ocular risk factors, including maternal pre-pregnancy BMI, preterm birth, and low birth weight, which showed strong associations with the development of amblyopia. These findings underscore the multifactorial nature of amblyopia, involving a complex interplay between genetic predisposition and environmental influences. Given the implications of these findings, it is imperative to incorporate routine amblyopia screening into pediatric care, particularly for populations at higher risk, to ensure timely diagnosis and effective management. This approach will aid in minimizing the long-term consequences of amblyopia and help children achieve their full visual and developmental potential. Early identification and intervention are crucial for optimal visual outcomes, emphasizing the need for preschool-based amblyopia screening programs.

## References

[1] Pai AS, Rose KA, Leone JF (2012). Amblyopia prevalence and risk factors in Australian preschool children. Ophthalmology.

[2] Williams C (2009). Amblyopia. BMJ Clin Evid.

[3] Jang JU, Park IJ (2015). The status of refractive errors in elementary school children in South Jeolla Province, South Korea. Clin Optom.

[4] (2006). The Apgar score. Pediatrics.

[5] Gupta D, Bhatnagar K, Kumar A, Kumar R (2019). Prevalence of amblyopia among school children. J Emerg Technol Innov Res.

[6] Ramkumar VA, Agarkar S, Mukherjee B (2016). Nasolacrimal duct obstruction: does it really increase the risk of amblyopia in children?. Indian J Ophthalmol.

[7] Gopal SK, Kelkar J, Kelkar A, Pandit A (2019). Simplified updates on the pathophysiology and recent developments in the treatment of amblyopia: a review. Indian J Ophthalmol.

[8] Wallace DK, Morse CL, Melia M, Sprunger DT, Repka MX, Lee KA, Christiansen SP (2018). Pediatric Eye Evaluations Preferred Practice Pattern®: I. Vision screening in the primary care and community setting; II. Comprehensive ophthalmic examination. Ophthalmology.

[9] Majumdar S, Tripathy K (2025). Hyperopia. http://www.ncbi.nlm.nih.gov/books/NBK560716/.

[10] Binayi Faal N, Ojaghi H, Sadeghieh Ahari S (2021). Paired opposite 4 mm clear corneal incisions on steep meridian during phacoemulsification. J Curr Ophthalmol.

[11] Flitcroft DI, He M, Jonas JB (2019). IMI - defining and classifying myopia: a proposed set of standards for clinical and epidemiologic studies. Invest Ophthalmol Vis Sci.

[12] Magdalene D, Bhattacharjee H, Choudhury M, Multani PK, Singh A, Deshmukh S, Gupta K (2018). Community outreach: an indicator for assessment of prevalence of amblyopia. Indian J Ophthalmol.

[13] Menon V, Chaudhuri Z, Saxena R, Gill K, Sachdev MM (2005). Profile of amblyopia in a hospital referral practice. Indian J Ophthalmol.

[14] Kalikivayi V, Naduvilath TJ, Bansal AK, Dandona L (1997). Visual impairment in school children in southern India. Indian J Ophthalmol.

[15] Murthy GV, Gupta SK, Ellwein LB, Muñoz SR, Pokharel GP, Sanga L, Bachani D (2002). Refractive error in children in an urban population in New Delhi. Invest Ophthalmol Vis Sci.

[16] Anjaneyulu K, Narendranath Reddy G (2015). Prevalence of amblyopia in children aged from 5-15 years in rural population Kurnool Dist. Andhra Pradesh, India. Int J Sci Res.

[17] (2008). Prevalence of amblyopia and strabismus in African American and Hispanic children ages 6 to 72 months the multi-ethnic pediatric eye disease study. Ophthalmology.

[18] Mocanu V, Horhat R (2018). Prevalence and risk factors of amblyopia among refractive errors in an Eastern European population. Medicina (Kaunas).

[19] Ganekal S, Jhanji V, Liang Y, Dorairaj S (2013). Prevalence and etiology of amblyopia in Southern India: results from screening of school children aged 5-15 years. Ophthalmic Epidemiol.

[20] Pascual M, Huang J, Maguire MG (2014). Risk factors for amblyopia in the vision in preschoolers study. Ophthalmology.

[21] Hendler K, Mehravaran S, Lu X, Brown SI, Mondino BJ, Coleman AL (2016). Refractive errors and amblyopia in the UCLA Preschool Vision Program; first year results. Am J Ophthalmol.

[22] Donahue SP (2006). Relationship between anisometropia, patient age, and the development of amblyopia. Am J Ophthalmol.

[23] Sapkota K, Pirouzian A, Matta NS (2013). Prevalence of amblyopia and patterns of refractive error in the amblyopic children of a tertiary eye care center of Nepal. Nepal J Ophthalmol.

[24] Robaei D, Rose KA, Kifley A, Cosstick M, Ip JM, Mitchell P (2006). Factors associated with childhood strabismus: findings from a population-based study. Ophthalmology.

[25] Pan CW, Qian DJ, Zhu H, Yu JJ, Liu H (2017). Apgar score and reduced vision in children aged 3 to 6 years. Graefes Arch Clin Exp Ophthalmol.

